# Chromosome-level Genome Assembly of Korean Long-tailed Chicken and Pangenome of 40 Gallus *gallus* Assemblies

**DOI:** 10.1038/s41597-024-04287-9

**Published:** 2025-01-11

**Authors:** Hanshin D. Shin, Wonchoul Park, Han-ha Chai, Youngho Lee, Jaehoon Jung, Byung June Ko, Heebal Kim

**Affiliations:** 1 Interdisciplinary Program in Bioinformatics, Seoul National University, Seoul, Republic of Korea; 2 Animal Genomics & Bioinformatics Division, National Institute of Animal Science, RDA 1500, Wanju, 55365 Republic of Korea; 3 Department of Agricultural Biotechnology and Research Institute of Agriculture and Life Sciences, Seoul National University, Seoul, Republic of Korea

**Keywords:** Genome assembly algorithms, Data publication and archiving

## Abstract

This study presents the first chromosome-level genome assembly of the Korean long-tailed chicken (KLC), a unique breed of Gallus gallus known as *Ginkkoridak*. Our assembly achieved a super contig N50 of 5.7 Mbp and a scaffold N50 exceeding 90 Mb, with a genome completeness of 96.3% as assessed by BUSCO using the aves_odb10 set. We also constructed a comprehensive pangenome graph, incorporating 40 Gallus gallus assemblies, including the KLC genome. This graph comprises 87,934,214 nodes, 121,720,974 edges, and a total sequence length of 1,709,850,352 bp. Notably, our KLC assembly contributed 1,919,925 bp of new sequences to the pangenome, underscoring the unique genetic makeup of this breed. Furthermore, in comparison with the pangenome, we identified 36,818 structural variants in KLC, which included 2,529 insertions, 27,743 deletions, and 6,546 of either insertions or deletions shorter than 1 kb. We also successfully identified pan-genome wide non-reference sequences. Our KLC assembly and pangenome graph provide valuable genomic resources for studying G. *gallus* populations.

## Background & Summary

The Korean long-tailed chicken (KLC), commonly referred to as “Ginkkoridak” in Korean, a breed distinguished by its notably long tail, represents a crucial element of Korea’s avian genetic legacy and occupies a significant role in worldwide biodiversity^1,2^. Remarkably, roosters at the age of three can extend their tail feathers up to 1.5 meters annually, while hens, although their tails are not as long as the roosters’, can still achieve a growth of up to 40 cm per year, which is relatively lengthy compared to other breeds^3,4^. Unfortunately, the KLC population experienced a significant reduction, especially during and subsequent to the Korean War. This decline was primarily due to the influx of foreign poultry breeds and changes in agricultural methods^2–4^. As of 2018, the KLC population had dwindled to approximately 450 individuals. This decline has highlighted the urgent need for detailed genetic research at the assembly level to understand and preserve the KLC’s unique genetic makeup, which is vital for maintaining biodiversity and has potential applications in various scientific fields.

**Fig. 1:**
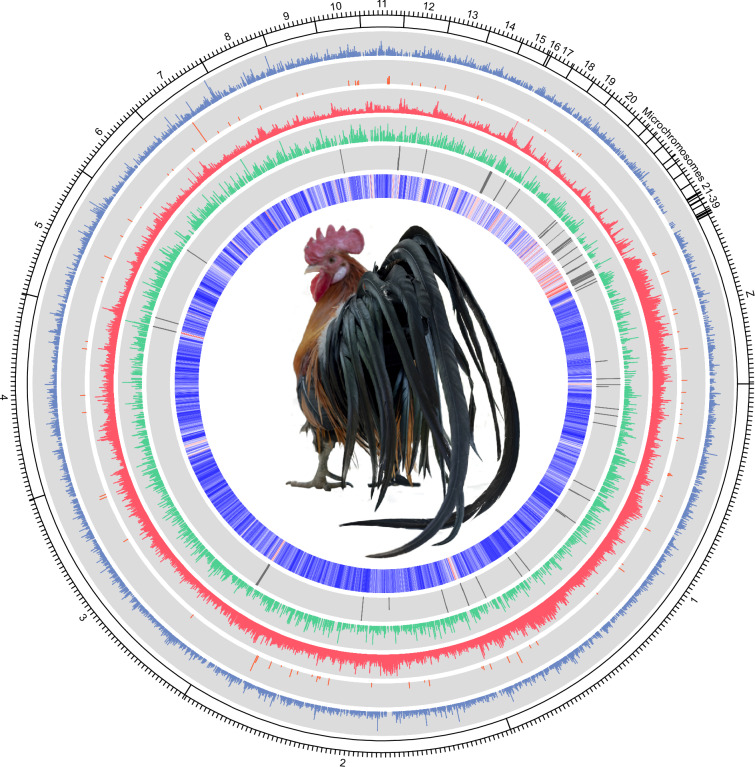
Circos plot of KLC. The circos plot was plotted using TBtools-II v.2.070.^22^ From the outer most track, each track represents: chromosomes including 39 autosomal chromosomes and sex chromosome Z, DNA TEs, LTRs, LINEs, SINEs, gaps and GC ratio, respectively. Each tick of the chromosome track represents 20,000 bp. Value of each element was calculated for every 10,000 bp window of the genome. For DNA TEs, LTRs, LINEs, SINEs and gaps, bar was plotted by taking the mean value of all 10,000 bp windows on every 100,000 bp and the global minimum and maximum values for every windows of genome were used for minimum and maximum height of the tracks (DNA TEs: 0, 1,936; LTRs: 0, 756; LINEs: 30,451, 0; SINEs: 0, 1,263; gaps: 0%, 1%). Chicken image is from Youm *et al*.^2^.

Here, we constructed a first chromosome-level genome assembly of KLC using a combination of PacBio long reads, 10x Genomic reads and Illumina short reads (Fig. 1). The assembly features a super contig N50 of 5.7 Mb and a scaffold N50 of over 90 Mb. The genome’s completeness, assessed using the aves_odb10 set, was confirmed with a BUSCO^5^ score of 96.3%, reflecting the thoroughness and accuracy of the assembly.

**Fig. 2:**
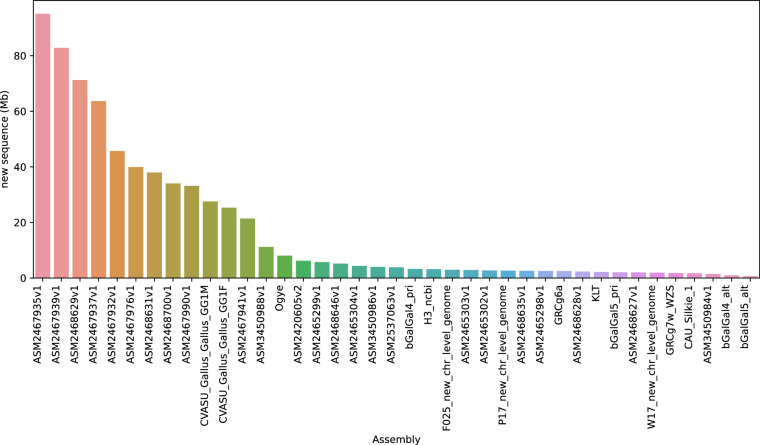
Contributing sequences to the pangenome. The graph depicts the sequence contributed by each sample arranged in order of the amount of sequence these assemblies provide.

Then, complementing this genome assembly, a comprehensive pangenome graph was constructed, integrating 40 Gallus gallus genome assemblies, including that of the KLC. This pangenome graph consisted of 87,934,214 nodes, 121,720,974 edges, sequence length of 1,709,850,352 and a mean degree (number of edges attached to a node) of 1.4. In total of 1,709,850,352 bp of sequence, the reference genome (GRCg7b)^6^ covered 1,041,122,857 bp of sequence, while our KLC assembly covered 1,919,925 bp of new sequences. (Fig. 2).

## Methods

### Sample collection and sequencing

A rooster of KLC was used in this study. Blood samples were gathered in compliance with the guidelines set by the National Institute of Animal Science (NIAS) in South Korea. All animal-related experimental procedures received approval from NIAS (approval number NIAS2018268). This study was conducted adhering to the ARRIVE guidelines.

Genomic sequencing was performed using multiple platforms to ensure comprehensive coverage. High-quality genomic DNA was extracted and sequenced using PacBio long-read technology, Illumina short-read sequencing with insert sizes of 280 bp and 550 bp, and 10x Genomics reads. The Illumina short-read sequencing achieved a depth of 19.661x for the 280 bp insert library and 5.909x for the 550 bp insert library. The 10x Genomics read sequencing provided a depth of 37.546x while the PacBio long-read sequencing generated a coverage depth of 88.183x. To summarize the sequencing data obtained from each platform, refer to Table 1, which provides detailed statistics including the platform used, tissue type, total number of reads, total bases, and SRA accession.

**Table 1:** Statistics of sequencing data; Platform, tissue, total bases, SRA accession.

Platform	Tissue	Reads	Total bases (bp)	SRA accession
Pacific Biosciences	Blood	8,892,027	103,500,936,727	SRX24958070
Illumina WGS (280 bp)	Spleen	483,460,306	73,002,506,206	SRX24958072
Illumina WGS (550 bp)	Spleen	275,595,838	41,614,971,538	SRX24958073
10x Genomics	Spleen	357,897,716	54,042,555,116	SRX24958071

#### Short read(PE) sequencing

 The quality of genomic DNA was assessed by agarose gel electrophoresis, and quantification was carried out using the Quant-IT PicoGreen assay (Invitrogen). Sequencing libraries were constructed following the guidelines of the TruSeq DNA Nano Library Prep Kit (Illumina, Inc., San Diego, CA, USA). In summary, the genomic DNA was fragmented using adaptive focused acoustic technology (AFA; Covaris). The resulting fragments were end-repaired to generate 5′-phosphorylated, blunt-ended double-stranded DNA. After end-repair, the DNA was size-selected through a bead-based approach. An ‘A’ base was then added to the 3′ ends, followed by the ligation of TruSeq indexing adapters. The final libraries were quantified via qPCR (using the KAPA Library Quantification Kit for Illumina platforms) and checked for quality using the Agilent Technologies 4200 TapeStation (Agilent Technologies). Paired-end sequencing was carried out on the HiSeq X platform (Illumina, San Diego, USA) at Macrogen (Seoul, South Korea).

#### Pacbio sequencing

 High-quality, high-molecular-weight DNA was required to generate size-selected SMRTbell templates of approximately 10 kb. DNA concentration was measured using a NanoDrop spectrophotometer (Thermo Scientific) and PicoGreen assay. All samples met the QC screening criteria. For PacBio Sequel sequencing, 5 µg of input genomic DNA was used to prepare the 10 kb library. For genomic DNA with a size distribution below 17 kb, the actual size distribution was determined using the Bioanalyzer 2100 (Agilent). If the apparent size exceeded 40 kb, the DNA was sheared using g-TUBE (Covaris Inc., Woburn, MA, USA) and purified with AMPurePB magnetic beads (Beckman Coulter Inc., Brea, CA, USA). A total of 10 µL of library was prepared using the PacBio DNA Template Prep Kit 1.0. SMRTbell templates were annealed using the Sequel Binding and Internal Ctrl Kit 3.0. Sequencing was carried out with the Sequel Sequencing Kit 3.0 and SMRT Cells 1 M v3 Tray. Data were captured from each SMRT cell using 600-minute movies on the PacBio Sequel sequencing platform (Pacific Biosciences) by Macrogen (Seoul, South Korea). Subsequent steps followed the PacBio Sample Net-Shared Protocol, available at http://pacificbiosciences.com/.

#### 10x Genomics

 The sequencing libraries were prepared according to the manufacturer’s instructions of Chromium Genome Library Kit (10x Genomics). Briefly, 40 kb or more of HMW genomic DNA were size selected using BluePippin(Sage Science) and 1 μg was used as input. Size selected HMW template gDNA was combined with 10x barcoded Gel bead to barcode DNA. The barcoded DNA underwent end-repair to generate 5′-phosphorylated, blunt-ended double-stranded DNA molecules. Afterward, a single ‘A’ base was added, followed by adapter ligation. The adapter-ligated DNA is amplified with an indexed primer to complete the chromium genome libraries with full-length adapters. Finally, the product is size selected with a bead-based method. The libraries were quantified following the qPCR Quantification Protocol Guide (KAPA Library Quantification kits for Illumina Sequencing platforms) and their quality was evaluated using the Agilent Technologies 4200 TapeStation D1000 ScreenTape (Agilent Technologies). Then we sequenced using the HiSeq (Illumina).

### Genome assembly

We initiated the process by filtering the raw Illumina short reads using NGSQCToolkit’s^7^ IlluQC module. Reads were retained only if at least 70% of their length exhibited a Phred quality score (Q score) of 20 or higher, which corresponds to a base call accuracy of 99%. Next, we employed Trimmomatic^8^ to remove adaptor sequences from the reads. After this step, we utilized fastQC^9^ to assess the quality of the trimmed reads, ensuring removal of any residual artifacts. Then SOAPec’s^10^ Corrector module was utilized to correct sequencing errors in paired-end reads with insert sizes of 280 and 550 base pairs. Finally, we utilized LoRDEC^11^ algorithm to correct sequencing errors specific to PacBio sequences. The genomic assembly was conducted in a hybrid fashion, combining short reads and long PacBio reads. Falcon^12^ generated primary contigs, Falcon-Unzip leveraged PacBio long reads and 10x Genomic Reads to create haplotigs, and GapCloser (Gap-DOES) utilized mate-pair reads to fill gaps. This integrated approach yielded a high-quality assembly with super contig N50 of 5.7 megabase pairs (Mbp) and a total of 1162 super contigs (Table 2: Statistics of contig-assembly before scaffolding). Purge_dups (v1.2.6)^13^ was used to remove haplotypic duplication and contig overlaps in the draft assembly.

**Table 2:** Statistics of contig assembly before scaffolding.

Statistics without reference	Draft primary contig assembly	Draft alternate contig assembly	Purged assembly
Number of contigs	1,162	1,162	662
Largest contig	28,796,376	28,780,808	28,796,376
Total length	1,061,949,247	1,062,049,020	1,021,991,062
N50	5,778,181	5,779,725	6,028,466
L50 contig count	47	47	44
GC (%)	42.27	42.27	42.04

After the removal of haplotypic duplication, our assembly was scaffolded using a reference-guided approach with RagTag (v2.1.0)^14^. Given the absence of the W chromosome in male chickens (Gallus gallus), which have ZZ sex chromosomes (females are ZW), we used GRCg7b^6^ (excluding the W chromosome) as a reference. The RagTag process was divided into two steps: ‘ragtag correct’ and ‘ragtag scaffold’ which corrects potential misassemblies by aligning our contig assembly and scaffolds the query contigs guided by the reference genome respectively. This resulted in 304 scaffolds including 39 chromosome-level scaffolds encompassing the Z chromosome, and 264 unplaced scaffolds (Table 3: KLC genome assembly statistics).

**Table 3:** Korean long-tailed chicken(KLC) genome assembly statistics.

Assembly statistics	Value
Genome size (bp)	1,023,165,347
Number of scaffolds	304
Number of chromosome-scale scaffolds	40
N50 of scaffolds (bp)	90,511,606
L50 of scaffolds	4
Chromosome-scale scaffolds (bp)	1,008,187,434
GC content of the genome (%)	42.04%
**BUSCO analysis**	
Library	aves_odb10
Complete	8,041 (96.43%)
Complete and single copy	8,012 (96.09%)
Complete and duplicated	29 (0.35%)
Fragmented	62 (0.74%)
Missing	235 (2.81%)

Then the PacBio long reads we employed for our assembly were aligned using minimap2 within the TGS-GapCloser^15^ framework for gap filling. The completed assembly of 39 chromosome-level scaffolds amounted to a total size of 995.29 Mb. The 39 chromosome-level scaffolds constituted 98.54% of the entire assembly, while the 14.98 Mb of the genome remains to be further studied (Table 4: Length of chromosome-level scaffolds).

**Table 4:** Length of Chromosome-level scaffolds.

Chromosome	Length	% of assembly
1	195,432,966	19.10081949
2	148,948,886	14.55765546
3	110,375,418	10.78764232
4	90,511,606	8.846234508
5	59,345,498	5.800186468
6	35,192,565	3.439577494
7	36,216,801	3.539682135
8	29,284,060	2.862104359
9	23,499,132	2.296709136
10	19,909,730	1.945895652
11	19,513,749	1.907194087
12	20,096,594	1.964158976
13	18,023,899	1.761582236
14	15,117,088	1.477482407
15	12,737,682	1.244928988
16	1,176,078	0.1149450578
17	10,679,723	1.043792485
18	11,299,519	1.104368813
19	10,034,254	0.9807069824
20	13,953,091	1.363718097
21	6,807,633	0.665350231
22	4,736,118	0.4628888199
23	5,818,881	0.5687136509
24	6,365,640	0.6221516413
25	2,932,729	0.2866329483
26	5,325,154	0.5204587915
27	5,718,884	0.5589403528
28	5,174,667	0.5057508071
29	436,614	0.0426728682
30	970,373	0.09484029173
31	1,572,772	0.1537163084
32	234,640	0.02293275478
33	2,109,227	0.2061472279
34	2,859,599	0.2794855209
35	745,058	0.07281892435
36	367,897	0.03595674942
37	123,200	0.01204106456
38	769,429	0.0752008463
39	192,591	0.01882305735
Z	76,830,679	7.509116608
Total	1,023,165,347	

### Repeat annotation of genome assembly

Repeat elements were identified with RepeatMasker v4.1.5^16^ using Dfam v3.7 library and RepBase (v 10/26/2018) using RMBlast. Approximately 10.28% of the genome was composed of repetitive elements. Retroelements, primarily Long Interspersed Nuclear Elements (LINEs), emerged as the dominant class, accounting for 7.14% of the genome. Short Interspersed Nuclear Elements (SINEs) were also observed, although they represent a smaller fraction. A detailed breakdown of repeat elements and their genomic proportions is provided in Table 5: Statistics of repetitive elements.

**Table 5:** Statistics of repetitive elements.

	# of elements	Total length (bp)	% of genome
SINE	4,079	583,994	0.06%
LINE	185,807	68,245,598	6.67%
LTR	26,810	13,416,239	1.31%
DNA transposons	30,563	10,249,430	1.00%
Unclassified	2,125	387,415	0.04%
Total interspersed repeats		92,882,676	9.08%
Small RNA	1,617	219,469	0.02%
Satellites	4,172	2,954,409	0.29%
Simple repeats	301,934	14,185,068	1.39%
Low complexity	53,708	3,045,313	0.30%
Total bases masked		113,131,366	11.06%

### Assessment of the chromosome-level genome assembly

The quality and completeness of the genome assembly were assessed using the Benchmarking Universal Single-Copy Orthologs (BUSCO)^5^ v5.5.0 software docker image, which evaluates the presence of evolutionarily informed expected gene content. Our analysis with the aves_odb10 lineage dataset revealed that 96.33% of the assessed orthologs were complete, indicating a high level of completeness in the assembly. Additionally, 0.008% were identified as fragmented, and 0.027% were missing, providing a clear metric of assembly quality. Furthermore, the assembly was evaluated with QUAST v5.2.0^17^ docker image for its contiguity and accuracy. Key metrics derived from QUAST included a contig N50 of 90.512 Mb, which is similar to the current reference genome, GRCg7b^6^. The largest contig assembled measured 195.43 Mb. The total number of contigs was 304, with 302 contigs being longer than 1 kb. The genome assembly also had a GC content of 42.04%, which is within the expected range for this species. These statistics underscore the robustness of our assembly process and the high quality of the genomic resource produced.

### Pangenome graph construction

To construct a comprehensive pangenome graph, we aggregated 40 available Gallus gallus genomes from the NCBI GenBank database, as of June 3rd, 2024. This collection encompassed a diverse range of assembly levels, featuring 21 chromosome-level assemblies, including the notable KLC assembly, along with 15 scaffold-level and 4 contig-level assemblies. For the establishment of a reference genome within this pangenome, we selected “GRCg7b”^6^, also recognized as bGalGal1b. This decision was informed by its status as the current species reference assembly in NCBI RefSeq, notable for its contig N50 value of 18.8 Mb. The GRCg7b assembly, renowned for its high-quality, fully annotated sequence, offers an extensive insight into the genomic architecture of the domestic chicken.

Minigraph–cactus pangenome pipeline^18^ with Docker image cactus v2.7.0 was used to build a pangenome because it enabled to incorporate not only chromosome-level assemblies, but also scaffold and contig-level assemblies to the pangenome. Due to different chromosomal structures among assemblies (Table 6: Chromosome structures of 40 assemblies), we had to manually run each step of the pipeline to retrieve all the macro and microchromosomes of Gallus gallus.

**Table 6:** Chromosome structures of 40 assemblies.

Assembly structure (Count of assemblies in the category)	Assembly name
1–39 + ZW (10 assemblies)	bGalGal1.mat.broiler.GRCg7b^6^
	bGalGal1.pat.whiteleghornlayer.GRCg7w_WZ (GCA_016700215.2)
	bGalGal5.pri (GCA_027408255.1)^27^
	bGalGal4.pri (GCA_027557775.1)^27^
	Ggswu (GCA_024206055.2)^28^
	P17_new_chr_level_genome (GCA_030849555.1)^29^
	F025_new_chr_level_genome (GCA_030849555.1)^29^
	W17_new_chr_level_genome (GCA_030914275.1)^29^
	H3_ncbi (GCA_030979905.1)^29^
	CVASU_Gallus_Gallus_GG1F (GCA_034769275.1)^29^
1–39 + Z (2 assemblies)	KLT (GCA_039997075.1)
	CVASU_Gallus_Gallus_GG1M (GCA_034769225.1)
1–38 + Z (1 assembly)	CAU_Silkie_1.0 (GCA_033088195.1)
1–28 + ZW (1 assembly)	Ogye1.0 (GCA_002798355.1)^30^
1–28, 30–33 + ZW (5 assemblies)	ASM2537063v1 (GCA_024653035.1)^31^
	ASM2465302v1 (GCA_024653025.1)^31^
	ASM2465299v1 (GCA_024652995.1)^31^
	ASM2465304v1 (GCA_024653045.1)^31^
	GRCg6a^32^
1–28, 30–33 + Z (2 assemblies)	ASM2465303v1 (GCA_024653035.1)^31^
	ASM2465298v1 (GCA_024652985.1)^31^
Scaffold level (15 assemblies)	bGalGal5.alt (GCA_027408225.1)
	bGalGal4.alt (GCA_027408255.1)
	ASM2468635v1 (GCA_024686355.1)^31^
	ASM2468646v1 (GCA_024686465.1)^31^
	ASM2468628v1 (GCA_024686285.1)^31^
	ASM2467976v1 (GCA_024679765.1)^31^
	ASM2467932v1 (GCA_024679325.1)^31^
	ASM2467990v1 (GCA_024679905.1)^31^
	ASM2468700v1 (GCA_024687005.1)^31^
	ASM2467941v1 (GCA_024679415.1)^31^
	ASM2468629v1 (GCA_024686295.1)^31^
	ASM2468631v1 (GCA_024686315.1)^31^
	ASM2467935v1 (GCA_024686355.1)^31^
	ASM2467939v1 (GCA_024679395.1)^31^
	ASM2467937v1 (GCA_024679375.1)^31^
Contig level (4 assemblies)	ASM2468627v1 (GCA_024686275.1)^31^
	ASM3450986v1 (GCA_034509865.1)
	ASM3450984v1 (GCA_034509845.1)
	ASM3450988v1 (GCA_034509885.1)

### Structural variants and genetic variation in KLC

To extract structural variants within the pangenome of Korean Long-Tailed (KLC) chicken, we employed the Minigraph-Cactus pangenome pipeline. This pipeline generates multi-sample VCF files containing genotypes relative to the pangenome graph. To isolate variants specific to the KLC sample, we adopted a targeted approach as outlined in Liao *et al*.^19^. Variants were filtered using a stringent criterion: those with a length difference between the reference and alternate alleles exceeding 50 base pairs were classified as deletions, while those where the alternate allele length surpassed the reference by 50 base pairs or more were classified as insertions.

**Fig. 3:**
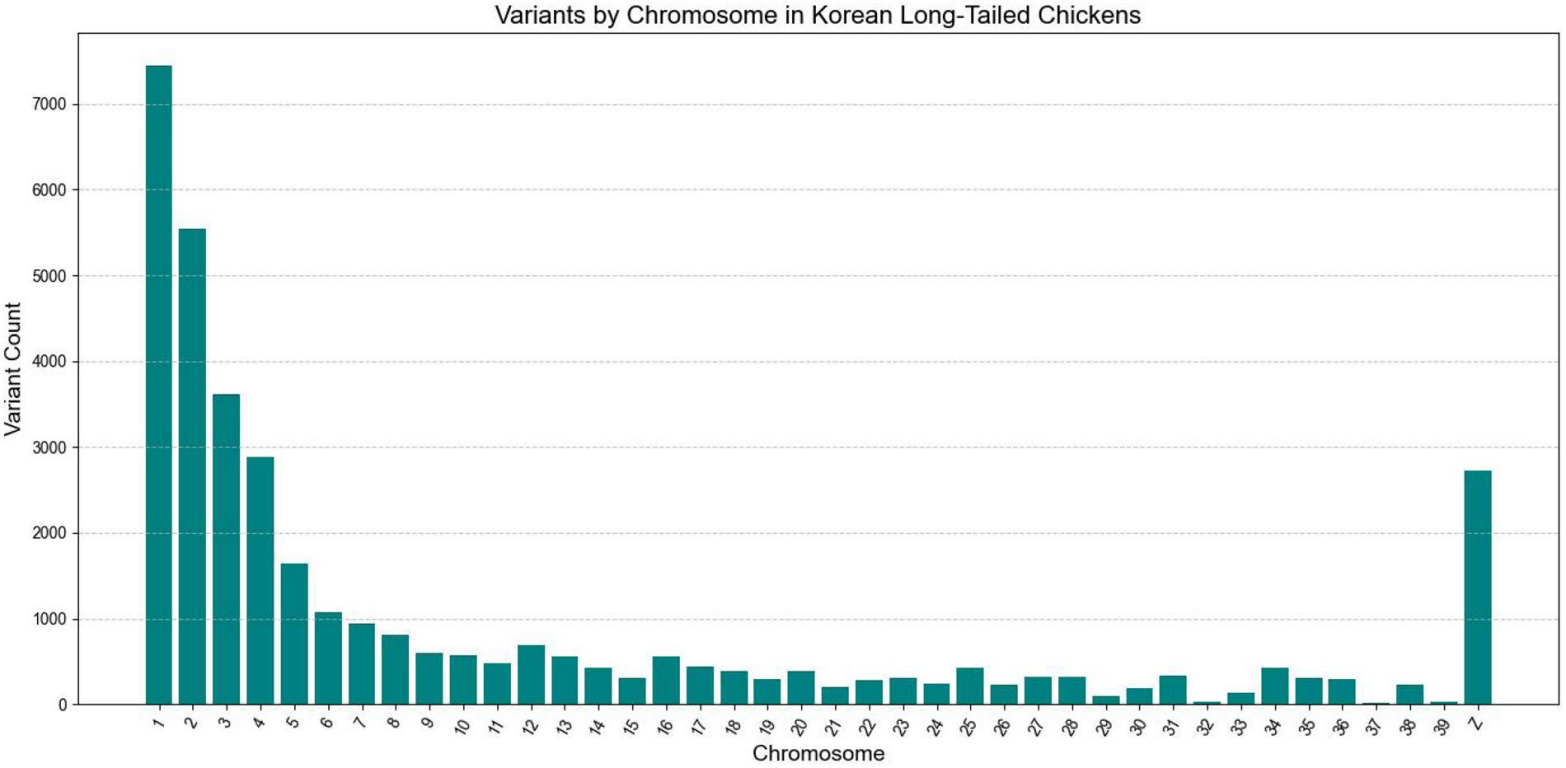
Variants by Chromosome in Korean Long-Tailed Chicken. This figure illustrates the distribution of genetic variants across the chromosomes of the Korean Long-Tailed Chicken (KLC). It highlights the frequency and types of variants, including insertions, deletions, and indels, across different chromosomes.

Additionally, we used the Variant Effect Predictor (VEP) from Ensembl^20^ to understand the genetic variations in KLC chicken. VEP identified the distribution of variant classes, showing a predominance of deletions (27,743), followed by indels (6,546) and insertions (2,529). In this analysis, insertions or deletions less than 1 kb were categorized as indels. VEP also provided the chromosomal distribution of these variants, as presented in Fig. 3.

### Pangenome graph and non-reference sequences

**Fig. 4:**
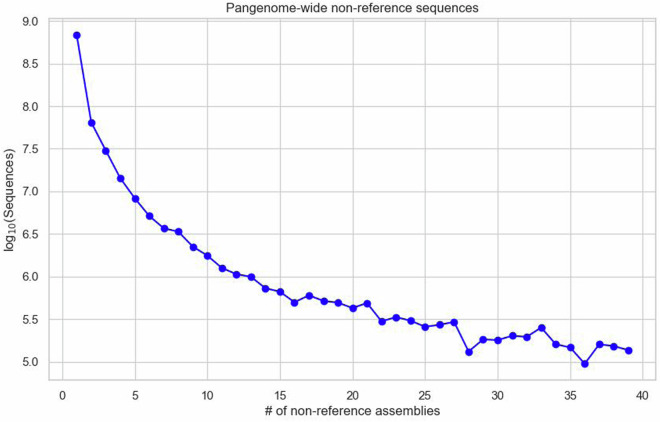
Pangenome-wide non-reference sequences. This figure presents a log-scale comparison of the total base pairs contributed by each assembly to the pangenome. It underscores the diversity and richness of the genomic content across different Gallus gallus assemblies, including the unique contributions from the KLC genome, thereby demonstrating the breadth and depth of the pangenome.

**Fig. 5:**
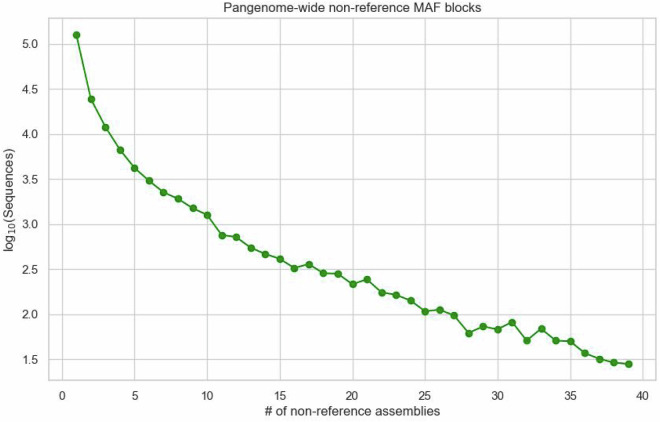
Pangenome-wide non-reference MAF blocks. Figure 5 provides a log-scale graphical representation of the number of assemblies versus the frequency of genomic MAF blocks appearing in the pangenome.

To quantify the unique sequences each sample added to the pangenome graph, we used a Python script that adapts the iterative approach described Rice and colleagues^21^. This script removes sequences covered by the reference assembly and iteratively eliminates sequences from the largest non-reference contributor until all samples are evaluated, effectively isolating novel sequences introduced by each genome. Additionally, to extract non-reference sequences from the pangenome graph, we converted the HAL output file from the Minigraph-Cactus pipeline^18^ into a MAF file using cactus-hal2maf tool. We filtered out blocks containing Ancestral, synthetic, or reference genome sequences (i.e., ‘Anc0’, ‘MINIGRAPH’, and ‘GRCg7b’) to extract non-reference alignments. The MAF files were used to determine the number of assemblies present and the total base pair counts in genomic blocks that do not align with reference genomes (non-reference blocks). The data regarding the base pairs in each assembly and the frequency of each genomic block appearance is presented in Figs. 4, 5, respectively. The script used for this analysis is available in the associated code repository.

## Data Records

The Korean long-tailed chicken assembly project has been deposited at DDBJ/ENA/GenBank under the accession JBBEWE01^22^.

This whole genome shotgun sequencing of Illumina short reads of 280 bp and 550 bp have been deposited at DDBJ/ENA/GenBank under the accession SRR29445729^23^ and SRR29445730^23^, respectively.

The PacBio sequencing data was deposited in the SRA at NCBI SRR29445732^23^.

The 10x Genomics read data was deposited in the SRA at NCBI SRR29445731^23^.

The variant data, which is including the structural variation (SV) from the pangenome analysis, have been deposited in the European Variation Archive (EVA) at EMBL-EBI under the project accession number PRJEB81219^24,25^.

The pangenome graph output (GFA file)^26^ is also uploaded on figshare

## Technical Validation

The integrity and purity of the KLC genomic DNA were rigorously evaluated to ensure high-quality sequencing. DNA degradation was monitored using agarose gel electrophoresis, and the purity of DNA samples was assessed using the NanoDrop spectrophotometer (Thermo Scientific). We accepted only DNA samples with an optimal OD260/280 ratio of 1.8–2.0 and an OD260/230 ratio greater than 2.0, ensuring minimal contamination and degradation.

The completeness of the KLC genome assembly was critically assessed using BUSCO v5.5.0 with the aves_odb10 data set. This evaluation demonstrated a high completeness score of 96.3%, indicating that the majority of avian core genes were successfully captured in our assembly. Furthermore, the assembly was subjected to additional quality checks using tools like QUAST v5.2.0^17^, which confirmed the contiguity and accuracy of our assembly process. A contig N50 value of 90.512 Mb, comparable to the current reference genome, was achieved, reflecting the robustness of our assembly.

## Data Availability

The following section details the versions, settings, and parameters of the software utilized: NGSQCToolkitv2.3: https://github.com/mjain-lab/NGSQCToolkit ragtag: https://github.com/malonge/RagTag vg stats -N -E -l -p 20 <gfa file>> <output txt> bcftools: view -a -l -s <sample name> cactus-hal2maf: <jobstore> <hal file> <maf file> --refGenome Anc0 --chunkSize 500000 QUAST v5.2.0; python quast.py <genome> BUSCO v5.5.0; busco -I <genome> -l aves_odb10 -m genome purge_dups v1.2.6: https://github.com/dfguan/purge_dups RepeatMasker: https://www.repeatmasker.org/ TBtools: https://github.com/CJ-Chen/TBtools-II
